# Supplementary material for: Development of cognition in corvids
Source: Anim Cogn. 2026 Jul 29;29(1):76. doi: 10.1007/s10071-026-02088-8 (PMC13722769; doi:10.1007/s10071-026-02088-8)
Supplement: Supplementary file 1 [file 10071_2026_2088_MOESM1_ESM.docx]

**SUPPLEMENTARY INFORMATION**

**Development of cognition in corvids**

Rachael Miller, Emma Claisse, Adam Timulak, Nicola S. Clayton

**S1 Table***. Overview of cognitive domains tested in relation to development/ ontogeny within corvids*. Core/ foundational domain = object permanence, caching, learning, motor self-regulation, perception. Physical cognition = casual reasoning, tool use/ manufacture. Social cognition = social learning, gaze following, social behaviour, communication. Physical/social cognition = object manipulation, exploration, neophobia and play

| **Corvid species** | **No. of papers** | **“Core” cognition** | **“Social” cognition** | **“Physical” cognition** | **References** |
| --- | --- | --- | --- | --- | --- |
|  | **Total % and no. of species tested per domain** | | | |  |
|  | 16 | 69% (11 species) | 56% (9 species) | 31% (5 species) |  |
| American crow, *Corvus brachyhyrhynchos* | 1 |  | 1 |  | Mates et al, 2014 |
| Azure-winged magpie, *Cyanopica cyanus* | 2 | 1 | 1 |  | Wang et al, 2021; Wang et al, 2023 |
| Black-billed magpie, *Pica hudsonia* | 1 | 1 |  |  | Clary et al, 2014 |
| Californian scrub-jay, *Aphelocoma californica* | 1 | 1 |  |  | Salwiczek et al, 2009 |
| Clark’s nutcracker, *Nucifraga columbiana* | 1 | 1 |  |  | Clary et al, 2014 |
| Canada jay, *Perisoreus canadensis* | 1 |  | 1 |  | Freeman et al, 2021 |
| Eurasian jay, *Garrulus glandarius* | 2 | 1 |  | 1 | Davidson et al, 2017; Zucca et al, 2007 |
| Common raven, *Corvus corax* | 21 | 10 | 16 | 1 | Auersperg et al, 2015; Boucherie et al, 2020; Beck et al, 2020; Braun et al, 2012; Bugnyar et al, 2007; Damini et al, 2025; Gallego-Abenza et al, 2020; Gallego-Abenza et al, 2022; Gallup et al, 2022; Jacobs et al, 2019; Kabadayi et al, 2017; Kenward et al, 2011; Loretto et al, 2020; Miller et al, 2015, 2016, 2025; Scheid and Bugnyar, 2008; Schloegl et al, 2007; Stowe et al, 2006; Wenig et al, 2021; Zeiträg and Osvath, 2023 |
| Carrion crow, *Corvus corone* | 5 | 4 | 3 |  | Hoffman et al, 2011; Miller et al, 2015; 2016; 2025; Wascher & Youngblood, 2025 |
| Western Jackdaw,  *Coloeus monedula* | 6 | 3 | 4 |  | Auersperg et al, 2015; Schwab et al, 2008; Scheid et al, 2008; Ujfalussy et al, 2012; von Bayern et al, 2007; Zandberg et al, 2014 |
| New Caledonian crow, *Corvus* *moneduloides* | 8 | 1 | 3 | 6 | Auersperg et al, 2015; Holzhaider et al, 2010a; 2010b; 2011; Hunt et al, 2007; Kenward et al, 2006; 2005; 2011 |
| Florida scrub-jay, *Aphelocoma coerulescens* | 2 | 2 | 1 |  | Bebus et al, 2016; Fuirst et al, 2020 |
| Hawaiian crow/ ʻAlalā, *Corvus hawaiiensis* | 1 |  |  | 1 | Greggor et al, 2020 |
| Large-billed crow, *Corvus macrorhynchos* | 1 |  | 1 |  | Obozova et al, 2018 |
| Pinyon jay, *Gymnorhinus cyanocephalus* | 1 | 1 |  |  | Stafford et al, 2006 |
| Rook, *Corvus frugilegus* | 1 |  |  | 2 | Tebbich et al, 2007 |

**S2 Table***. Overview of abilities tested in relation to development/ ontogeny within corvids*. * = social behaviour includes anti-predator behaviour, social structure, social foraging, yawning. + = learning includes spatial, associative and reversal learning

| **Corvid species** | **No. of papers** | **Object permanence** | **Caching** | **+ Learning** | **Motor self-regulation** | **Perception** | **Neophobia/ exploration** | **Social Learning** | **Object manipulation and play** | **Gaze Following** | *** Social Behaviour** | **Communication** | **Causal reasoning** | **Tool use/ manufacture** | **References** |
| --- | --- | --- | --- | --- | --- | --- | --- | --- | --- | --- | --- | --- | --- | --- | --- |
| American crow, *Corvus brachyhyrhynchos* | 1 |  |  |  |  |  |  |  |  |  |  | 1 |  |  | Mates et al, 2014 |
| Azure-winged magpie, *Cyanopica cyanus* | 2 | 1 |  |  |  |  |  |  |  |  |  | 1 |  |  | Wang et al, 2021; Wang et al, 2023 |
| Black-billed magpie, *Pica hudsonia* | 1 |  |  |  |  | 1 |  |  |  |  |  |  |  |  | Clary et al, 2014 |
| Californian scrub-jay, *Aphelocoma californica* | 1 | 1 | 1 |  |  |  |  |  |  |  |  |  |  |  | Salwicxek et al, 2009 |
| Clark’s nutcracker, *Nucifraga columbiana* | 1 |  |  |  |  | 1 |  |  |  |  |  |  |  |  | Clary et al, 2014 |
| Canada jay, *Perisoreus canadensis* | 1 |  |  |  |  |  |  |  |  |  | 1 |  |  |  | Freeman et al, 2021 |
| Eurasian jay, *Garrulus glandarius* | 2 | 1 |  |  |  |  |  |  |  |  |  |  | 1 |  | Davidson et al, 2017; Zucca et al, 2007 |
| Common raven, Corvus corax | 21 | 1 | 7 | 1 | 1 |  | 3 | 1 | 4 | 2 | 5 | 1 |  | 1 | Auersperg et al, 2015; Boucherie et al, 2020; Beck et al, 2020; Braun et al, 2012; Bugnyar et al, 2007; Damini et al, 2025; Gallego-Abenza et al, 2020; Gallup et al, 2022; Kabadayi et al, 2017; Miller et al, 2015, 2016, 2025; Schloegl et al, 2007; Stowe et al, 2006; Wenig et al, 2021; Zeiträg and Osvath, 2023; Jacobs et al, 2019; Kenward et al, 2011; Scheid and Bugnyar, 2008; Gallego-Abenza et al, 2022; Loretto et al, 2020 |
| Carrion crow, Corvus corone | 5 | 1 | 3 |  |  |  | 3 |  | 1 |  |  | 1 |  |  | Hoffman et al, 2011; Miller et al, 2015; 2016; 2025; Wascher & Youngblood, 2025 |
| Western Jackdaw, Coloeus  monedula | 6 | 1 | 1 | 1 |  |  |  | 1 | 2 |  | 1 | 1 |  |  | Auersperg et al, 2015; Schwab et al, 2008; Scheid et al, 2008; Ujfalussy et al, 2012; von Bayern et al, 2007; Zandberg et al, 2014 |
| New Caledonian crow, Corvus moneduloides | 8 |  |  | 1 |  |  |  | 1 | 1 |  | 1 |  |  | 6 | Auersperg et al, 2015; Holzhaider et al, 2010a; 2010b; 2011; Hunt et al, 2007; Kenward et al, 2006; 2005; 2011 |
| Florida scrub-jay, Aphelocoma coerulescens | 2 |  | 1 | 1 |  |  | 1 |  |  |  |  |  |  |  | Bebus et al, 2016; Fuirst et al, 2020 |
| Hawaiian crow/ ʻAlalā, Corvus hawaiiensis | 1 |  |  |  |  |  | 1 |  |  |  |  |  |  |  | Greggor et al, 2020 |
| Large-billed crow, Corvus macrorhynchos | 1 |  |  |  |  |  |  |  |  |  | 1 |  |  |  | Obozova et al, 2018 |
| Pinyon jay, Gymnorhinus cyanocephalus | 1 |  |  | 1 |  |  |  |  |  |  |  |  |  |  | Stafford et al, 2006 |
| Rook, Corvus frugilegus | 1 |  |  |  |  |  |  |  |  |  |  |  | 1 | 1 | Tebbich et al, 2007 |

**S3 Table***. Papers from coded review output with corresponding tested species, specific cognitive abilities tested, developmental trajectories, outcome measures and environmental effects.* Core/ foundational domain = object permanence, caching, learning, motor self-regulation, perception. Physical cognition = casual reasoning, tool use/ manufacture. Social cognition = social learning, gaze following, social behaviour, communication. Physical and social cognition = object manipulation, exploration, neophobia and play. Social behaviour includes anti-predator behaviour, social structure, social foraging, yawning. Learning includes spatial, associative and reversal learning

| **Paper** | **Species** | **Cognitive domain** | **Specific ability tested** | **Life stages tested** | **Developmental trajectory** | **Outcome measures** |
| --- | --- | --- | --- | --- | --- | --- |
| Holzhaider et al, 2010a | New Caledonian crow | Physical | Tool use | Juvenile, adult | Improves with age | Object manipulation duration |
| Holzhaider et al, 2010b | New Caledonian crow | Physical | Tool use/ manufacture | Juvenile | Improves with age | Frequency of tool use events |
| Hunt et al, 2007 | New Caledonian crow | Physical | Tool use | Fledging, adult | Not analysed | Trials to criterion |
| Kenward et al, 2011 | New Caledonian crow, Common raven | Physical | Tool use | Fledging, juvenile | Improves with age | Object manipulation duration |
| Kenward et al, 2005 | New Caledonian crow | Physical | Tool use | Juvenile | Not analysed | Number of times tool used in attempt of food retrieval |
| Tebbich et al, 2007 | Rook | Physical | Tool use | Juvenile | Not analysed | Trials to criterion |
| Kenward et al, 2006 | New Caledonian crow | Physical | Tool use | Fledging, juvenile | Improves with age | Object manipulation duration, object choice |
| Von bayern et al, 2007 | Jackdaw | Social, physical | Food/object sharing | juvenile | Declines with age | Foraging behaviour |
| Miller et al, 2025 | Common raven,  Carrion / hooded crow | Social, physical, core | Exploration, caching | Fledging, juvenile, subadult | Improves with age | Frequency of object interaction |
| Miller et al, 2015 | Common raven,  Carrion / hooded crow | Social, physical, core | Exploration, caching | Fledging, juvenile, subadult | Declines with age | Frequency of object interaction |
| Miller et al, 2016 | Common raven,  Carrion / hooded crow | Social, physical, core | Exploration, caching | fledging, juvenile, subadult | No pattern | Frequency of object interaction, caching frequency |
| Stöwe et al, 2006 | Common raven | Social, physical | Exploration, object manipulation | Juvenile | Stable | Object manipulation duration, frequency of object interaction-s |
| Beck et al, 2020 | Common raven | Core | Caching | Juvenile | No pattern | Frequency of caching trips, cache probability, cache distance |
| Fuirst et al, 2020 | Florida scrub jay | Core | Caching | First year, second year, experience-d adult | Stable | Condition of cache sites, position of cache sites |
| Auersperg et al, 2015 | Common raven, jackdaw, New Caledonian crow (other species: red-shouldered macaw, black-headed caique, Goffin’s cockatoo) | Social, physical | Object manipulation, play | Juvenile, subadult-juvenile, adult | Not analysed | Frequency of object combinations |
| Wenig et al, 2021 | Common raven | Social, physical | Play | Juvenile | Stable | Object manipulation duration, frequency of object manipulat-ions |
| Damini et al, 2025 | Common raven | Social | Anti-predator responses | Mixed ages | improves with age | General behaviour |
| Schwab et al, 2008 | Jackdaw | Social | Social learning | Fledging, juvenile, subadult | Improves with age | Affiliation behaviour, object manipulation duration |
| Freeman et al, 2021 | Canada jay | Social | Social status | Nestling, juvenile | Stable | Physiology |
| Wang et al, 2023 | Azure-winged magpie | Social | Communication, vocal repertoire | Nestling, juvenile, adult | Stable | General behaviour |
| Gallup et al, 2022 | Raven | Social | Contagious yawning | Juvenile | No pattern | General behaviour |
| Braun et al, 2012 | Common raven | Social | Social grouping | Juvenile, subadult, adult | Not analysed | General behaviour |
| Obozova et al, 2018 | Large-billed crow | Social | Social interactions | Fledging then juvenile | U-shaped | General behaviour |
| Schloegl et al, 2007 | Common raven | Social | Gaze following | Nestling, fledging | Improves with age | Gaze following |
| Zeiträg and Osvath, 2023 | Common raven | Social | Gaze following | Fledging, juvenile | Improves with age | Gaze following |
| Holzhaider et al, 2011 | New Caledonian crow | Social | Social structure | Juvenile, adult | Not analysed | General behaviour inc. foraging behaviour |
| Wascher & Youngblood, 2025 | Carrion /hooded crow | Social | Communication | Juvenile, adult | Declines with age | Call sequences in response to general behaviour |
| Boucherie et al, 2020 | Common raven | Social | Social behaviour | Juvenile | Not analysed | General behaviour and frequency of object manipulations |
| Gallego‐Abenza et al, 2020 | Common raven | Social | Social foraging behaviour | Juvenile, subadult, adult | Improves with age | Foraging behaviour |
| Gallego-Abenza et al, 2022 | Common raven | Social | Social attention/recognition | Juvenile | Not analysed | General behaviour in response to calls |
| Loretto et al, 2020 | Common raven | Social | Social learning | Juvenile | Not analysed | Trials to criterion, type of contact with box |
| Mates et al, 2014 | American crow | Social | Communication | Subadult, adult | No pattern | Foraging behaviour in response to calls, general behaviour in response to calls |
| Zandberg et al, 2014 | Jackdaw | Social | Call recognition/discrimination | Nestling | Improves with age | General behaviour in response to calls |
| Bugnyar et al, 2007 | Common raven | Core | Caching, object permanence | Fledgling, juvenile | Improves with age | General behaviour |

**S4 Table***. Uzgiris & Hunt Scale 1 task / Object permanence development across corvids and parrots.* *Age in days at which last task of the OP stage was completed (so in task 5-9, age at which task 9 was completed). Azure-winged magpies excluded because even though subjects were reported to be juveniles, the study did not include developmental timing

| **Species** | **Sample size** | **Estimated fledging age**  **(days)** | **Stage 3**  **Task 3** | **Stage 4**  **Task 4** | **Stage 5**  **Tasks**  **5-9** | **Stage 6**  **Tasks 10-14** | **Stage 6**  **Task 15** | **“A-not-b” errors** | **Reference** |
| --- | --- | --- | --- | --- | --- | --- | --- | --- | --- |
| California Scrub-jay | 15 | 19 | g2: 28  g1: 32 | 35 | Not tested | Not tested | Not tested | Not tested | Salwiczek et al, 2009 |
| Eurasian jay  (mean age; younger jays’ group) | 9 total  Younger group: 5  Older group: 4 | 20 | 31 | 37 | 41 | 47 | 47 | No errors | Zucca et al, 2007 |
| Eurasian magpie  (mean age) | 11 | 25 | 32 | 57 | 107 | 180 | Did not pass | No errors | Pollok et al, 2000 |
| Common raven  (median ages, estimate based on weeks postfledging, two groups at different facilities, UVM and KLF) | 18 | 40 | UVM: 47  KLF: 47 | UVM: 54  KLF: 54 | UVM:  89  KLF: 89 | UVM: 110  KLF: 110 | UVM: did not pass  KLF: 110 | Errors present | Bugnyar et al, 2007 |
| Carrion crow  (median ages) | 5 | 32 | 37 | 42 | 67 | 71 | Did not pass | 5/5 subjects made errors | Hoffmann et al, 2011 |
| Jackdaw  (mean ages) | 19 | 26-31 | 36.6 | 41 | 61 | 74.3 | 80.7 | 2/8 subjects made errors | Ujfalussy et al, 2012 |
| Grey parrot | 1 | 70-84 | 63 days | 112 days | 140 days | 154 days | 147 days* | Errors present | Pepperberg et al, 1997 |
| Kakariki  (Parent-raised vs. hand-raised groups) | 11 total  Parent-reared group: 6  Hand-reared group: 5 | 35-49 | PR: 63 days  HR: 56 days | PR: 108 days  HR: 73 days | PR: 150 days  HR: 133 days | PR: 164 days  HR: 161 days | PR: 164 days  HR: 161 days | Errors present | Funk, 1996 |

**S5 Table.** List of 15 object permanence tasks from Uzgiris and Hunt’s (1975) Scale 1 with associated Piagetian stages. As different studies used different materials, descriptions are conceptual. Stages 4, 5 and 6 can also be further divided into substages 4a, 4b, 5a, 5b, 6a and 6b. Table adapted from Cornero & Clayton (2025).

| **OP Stage** | **Task** | **Task description** |
| --- | --- | --- |
| 2 | 1 | Following a moving object, in a 180-degree arc. |
| 2 | 2 | Noticing the disappearance of a moving object behind a barrier. |
| 3 | 3 | Finding an object that is partially covered. |
| 4 | 4 | Finding an object that is fully covered. |
| 5 | 5 | Finding an object that is completely covered, in two places. Checks for “A-not-B” error. |
| 5 | 6 | Finding an object covered in two places, alternately. |
| 5 | 7 | Finding an object covered in three places. |
| 5 | 8 | Finding an object after successive visible displacements. |
| 5 | 9 | Finding an object under three superimposed occluders. |
| 6 | 10 | Finding an object following one invisible displacement. |
| 6 | 11 | Finding an object following one invisible displacement with two occluders – checks for “A-not-B” error. |
| 6 | 12 | Finding an object following one invisible displacement with two occluders, alternately. |
| 6 | 13 | Finding an object following one invisible displacement with three occluders. |
| 6 | 14 | Finding an object following a series of invisible displacements. |
| 6 | 15 | Finding an object following a series of invisible displacements with evidence of representation. |

**S6 Table***. Taxonomic distribution of developmental studies on object permanence, gaze following and tool use in non-human animals.* For the cross-species comparison table, we did not conduct a systematic literature review. Instead, we used existing reviews of object permanence, gaze following and tool use to identify representative studies across taxa and then screened these sources for papers that focused on ontogenetic development or explicitly mentioned using multiple juvenile subjects (Gómez, 2005; Jaakkola, 2014; Meulman et al, 2013; Zeiträg et al, 2022). Citations provided for each species are thus illustrative rather than exhaustive – for example, although numerous studies examine chimpanzee tool-use ontogeny in different populations, a single representative example was included in this table.

| **Species tested *developmentally* or as juveniles** | **Object permanence** | **Gaze following** | **Tool use (habitual)** |
| --- | --- | --- | --- |
| Corvids | Eurasian magpie (Pollok et al, 2000),  Eurasian jay (Zucca et al, 2007).  Common raven (Bugnyar et al, 2007),  Carrion crow (Hoffmann et al, 2011),  Jackdaw (Ujfalussy et al, 2012),  California scrub-jay (Salwiczek et al, 2009),  Azure-winged magpie (Wang et al, 2021) | Common raven | New Caledonian crow (Kenward et al, 2006) |
| Parrots | Grey Parrot *Psittacus erithacus* (Pepperberg et al, 1997),  Kakariki *Cyanoramphus auriceps* (Funk, 1996) | [Grey parrot](https://link.springer.com/article/10.1007/s10071-008-0163-2) (Giret et al, 2008) |  |
| Other birds | [Domestic chicken](https://elifesciences.org/articles/67208) *Gallus gallus domesticus* (Szabó et al, 2022) | [Greylag goose](https://link.springer.com/article/10.1007/s10071-011-0381-x), *Anser anser* (Kehmeier et al, 2011),  [Bobwhite quail](https://pmc.ncbi.nlm.nih.gov/articles/PMC2764337/), *Colinus virginianus* (Jaime et al, 2009),  [Domestic chicken](https://www.sciencedirect.com/science/article/pii/S0166432806006231), *Gallus gallus domesticus* (Rosa Salva et al, 2007) | [Woodpecker finch](https://royalsocietypublishing.org/rspb/article-abstract/268/1482/2189/71004/Do-woodpecker-finches-acquire-tool-use-by-social?redirectedFrom=fulltext), *Camarhynchus pallidus* (Tebbich et al, 2001),  [Green-backed heron](https://onlinelibrary.wiley.com/doi/abs/10.1111/j.1474-919X.1986.tb02677.x) *Butorides sp*. (Higuchi, 2008) |
| Great Apes | Chimpanzee, *Pan troglodytes* (Wood et al, 1980),  [Orangutan](https://www.eva.mpg.de/documents/AmericanPsychologicalAss/Call_Object_JCompPsych_2002_1556106.pdf), *Pongo pygmaeus* (Call, 2001),  [Gorilla](https://www.taylorfrancis.com/chapters/edit/10.4324/9781315808055-5/early-sensorimotor-development-gorilla-giovanna-spinozzi-francesco-natale), *Gorilla gorilla* (Spinozzi & Natale, 2019) | [Chimpanzee](https://psycnet.apa.org/record/2001-00389-007), (Tomasello et al, 2001),  [Bonobo](https://research.ebsco.com/c/dvoqdt/search/details/5ceam423of), *Pan paniscus* (Bräuer et al, 2005),  [Gorilla](https://research.ebsco.com/c/dvoqdt/search/details/5ceam423of) (Bräuer et al, 2005),  [Orangutan](https://research.ebsco.com/c/dvoqdt/search/details/5ceam423of) (Bräuer et al, 2005) | [Chimpanzee](https://onlinelibrary.wiley.com/doi/abs/10.1002/ajpa.24125) (Musgrave et al, 2020), [Orangutan](https://www.cambridge.org/core/journals/evolutionary-human-sciences/article/ontogeny-%5B%E2%80%A6%5Dehaviour-in-wild-orangutans/81CE0FC52D716D58A20BC08AFC0D5752) (Schuppli et al, 2021) |
| Other primate | [Japanese Macaque](https://www.researchgate.net/publication/16013358_Cognitive_development_in_a_Japanese_macaque_Macaca_fuscata), *Macaca fuscata* (Antinucci et al, 1982),  [Rhesus macaque](https://www.researchgate.net/publication/232431138_Piagetian_object_permanence_in_the_infant_rhesus_monkey), *Macaca mulatta* (Wise et al, 1974)*,*  Crab-eating macaque *Macaca fascicularis* (Schino et al, 1990),  Tufted capuchin *Cebus apella* (Schino et al, 1990) | [Southern pig-tailed macaque,](https://pubmed.ncbi.nlm.nih.gov/11095722/) *Macaca nemestrina* (Ferrari et al, 2000),  [Rhesus macaque,](https://psycnet.apa.org/record/2001-00389-007) *Macaca mulatta* (Tomasello et al, 2001),  [Barbary macaque,](https://onlinelibrary.wiley.com/doi/10.1111/j.1467-7687.2010.00956.x) *Macaca sylvanus* (Teufel et al, 2010),  [Diana’s monkey](https://pdf.sciencedirectassets.com/272524/1-s2.0-S0003347200X02308/1-s2.0-S0003347204002398/main.pdf?X-Amz-Security-Token=IQoJb3JpZ2luX2VjEBsaCXVzLWVhc3QtMSJIMEYCIQCplNmNzW58WwNzqyp7BJ28wFlx8TtjkjrSpJikDI5HVQIhAOV6tA6NPOqj%2BM%2FZNjSmO%2BMbjd5i26sCEhVO%2BQ6qCC8kKrwFCOT%2F%2F%2F%2F%2F%2F%2F%2F%2F%2FwEQBRoMMDU5MDAzNTQ2ODY1IgyHB4SJr09L0ahTuesqkAWNZ1qeXb6tiMwusKXHdZSYERQs8X%2FfFL72t7yx5qFhL%2FMzAc4JWYulgenzApF6tlulG1WzZ7E48awKg1Nahqz6XJ%2FyE1k3BVFed7ZvKRsGg%2Bp5XohzSNZHAwtk2mDTwdFDf5ON0k1HCMft9gU0Xaj4I%2B5gJgstmearfw5zPjfsR717fQfeMj0kyL0YtNp4NemzqQDiedl%2B4PjfqtL53yO04aV3bXmS6CwTpTlcLFKfoNKgjwkG1EqjD%2FsHvFQfUt%2FDM27hQNctQXxe1LkjHI5aTnjfbtJFuyXit5eSWdtsS%2FsZN41felLcRyNyHkKA2L5cQBMTRGY%2FV1tfmbTOq%2FFORRkt2VYwRksvfWvotkNDIQ2jt%2Fnx1aq9VqBKkcd%2B2rVfMtOv0FLD5ea6S9ZQqJXRf9t%2FIIalxWLrZQSAq%2F3BwCpMbkWtUqHppAZ%2FMRQ77PHRUSR2Wcow8HLfRcCxsrtL%2FVHcuLSTIDuZLZjU1TCXdPxrVjCXJ72ZPBOmfrOwYIQo%2FNg7QupNRQslSjrL2%2BI9GoCQS5h65CydRlyJA9uh8Fp4T53dNyOMdYx1YvTsSRI4hQVyHjmfK2on1wxdok%2FHRCSDHGrSR4dstviDJw5IaVcsRMjjDj38ze9Y%2Bw0BQzD%2FH%2BurPvRWwTle4%2B9i2dpyZ%2BQ0uvQjIbajpoTy8mzt4TPk50xd9sVZDa1blquW2jz8Rj3ZE7viAKcBsNNeVZZG6eUnodkqrhSCnXqSvyv58ESLHCVv0J7X6ws2Hpn9oz3%2BGMan%2F9z%2BkI6iRQVXPVqflAi%2FettSWave8%2BeAyyViCgTfqaezILkb8mpc%2BCefmQrY3OHQg3J0YTEs%2FMDIhpfhK4qQXJDSoltJ9nY73qHWYDDc2snLBjqwAUr7JoXyMRedQpBfFypiumr14LBHCL9RQ%2FJ9IHNv%2BEPy7UJhQSTaZZIoY5jQrCvYDVm689H76srn2%2Bynj2IR6YCB0sFx8uw6D8L9541spqRXgmDrQyMtEKosZIksDqY5QU0Ou2aKtc6LGQlHVnq5nLdrx9QSYGyitlZ3wc0rhQZFmxX%2BzGBQAucxO3%2Fh1yaYdrzJAcwSy9YMtO2o%2FTsD2mOcw%2FWioBL5IHtuKdUx6CoJ&X-Amz-Algorithm=AWS4-HMAC-SHA256&X-Amz-Date=20260122T192054Z&X-Amz-SignedHeaders=host&X-Amz-Expires=300&X-Amz-Credential=ASIAQ3PHCVTYUHV2GQ2S%2F20260122%2Fus-east-1%2Fs3%2Faws4_request&X-Amz-Signature=2091b9c5c14dd60f6c2b558675cf2e830687f5e3146ed3d65a2b217ed46eb018&hash=f3f55143595189c79d73ea4ebb5fe5e63a2ddaee35cb65fcaf5e6e07d4ee6c58&host=68042c943591013ac2b2430a89b270f6af2c76d8dfd086a07176afe7c76c2c61&pii=S0003347204002398&tid=spdf-45bd6f72-814f-41a0-9760-f3f4f8e66a7e&sid=d87edfd3113860410819056112234a39cac5gxrqb&type=client&tsoh=d3d3LnNjaWVuY2VkaXJlY3QuY29t&rh=d3d3LnNjaWVuY2VkaXJlY3QuY29t&ua=01065701505452585602&rr=9c21694d7e07654c&cc=gb), *Cercopithecus diana diana* (Scerif et al, 2004),  [Cotton top tamarin](https://psycnet.apa.org/fulltext/2002-00850-001.html), *Saguinus oedipus* (Neiworth et al, 2002),  [Olive baboon](https://onlinelibrary.wiley.com/doi/10.1002/ajp.22580), *Papio anubis* (Parron & Meguerditchian, 2016) | [Tufted capuchin](https://onlinelibrary.wiley.com/doi/abs/10.1002/ajp.23251?casa_token=Lx9iFCU8zzgAAAAA:_KNAUOUcRg6Dcny37THEr05efs54LvKxd3aodWQbCKdM2A8xJLpKpw9P-zONdaOsYovBtkAf5fz5CIo), *Sapajus libidinosus* (Falótico et al, 2021),  Bearded capuchin *Cebus libidinosus* (Mannu & Ottoni, 2009),  [Cottontop tamarin](https://www.sciencedirect.com/science/article/pii/S000334720293068X), *Saguinus oedipus* (Hauser et al, 2002),  [Long-tailed macaque](https://psycnet.apa.org/fulltext/2017-12252-001.html), *Macaca fascicularis* (Tan, 2017) |
| Other mammals | [Domestic dog](https://psycnet.apa.org/buy/1994-43961-001), *Canis familiaris* (Gagnon & Doré, 1994),  Domestic cat, *Felis catus* (Dumas & Doré, 1989) | [Domestic dog](https://www.science.org/doi/full/10.1126/science.1072702) (Hare et al, 2002),  Wolf, *Canis lupus* (Range & Virányi, 2011),  Silver fox, *Vulpes vulpes* (Hare et al, 2005),  [Dingo](https://link.springer.com/article/10.1007/s10071-009-0287-z), *Canis dingo* (Smith & Litchfield, 2009),  [Domestic goat,](https://www.sciencedirect.com/science/article/pii/S0003347204003264?via%3Dihub) *Capra hircus* (Kaminski et al, 2005),  [Domestic pig](https://brill.com/view/journals/beh/138/11-12/article-p1337_2.xml), *Sus scrofa domesticus* (Byrne et al, 2001) | Sea otter, *Enhydra lutis* (Payne & Jameson, 1984),  Bottlenose dolphin, *Tursiops sp*. (Mann et al, 2008) |
| Other |  | [Leopard gecko,](https://link.springer.com/article/10.1007/s10071-018-1230-y) *Eublepharis macularius* (Simpson & O’Hara, 2018) |  |

**S1 Text.** *Coding definitions for systematic review*.

**A. Study metadata**

| **Field** | **What it captures** | **Coding / format** | **Example** |
| --- | --- | --- | --- |
| Study_ID | Unique identifier | FirstAuthor_Year_Journal_ShortSpecies | Clayton_1997_JExpPsych_Rook |
| Full_reference | Citation with APA | Text | Clayton NS & Krebs JR (1997) |
| Publication_year | Year | 4-digit integer | 1997 |
| Journal_name | Outlet type | free text | Animal Cognition |
| Country | Study country (where data collection took place; use full name) | free text | United Kingdom, United States of America |
| Setting | Broad context where data collection occured | lab, zoo, field, sanctuary, mixed | lab, zoo |

**B. Species & life-history**

| **Field** | **What it captures** | **Coding / format** | **Example** |
| --- | --- | --- | --- |
| Species | Scientific name | free text | *Corvus corax* |
| Common_name | Common name | free text | Common raven |
| Corvid_subfamily | Taxonomic subgroup | e.g. Corvinae, Cyanocoracinae, Cissinae, Pyrrhocorax, Perisoreinae | Corvinae |
| Wild_vs_captive_origin | Background of subjects | captive-bred, wild-bred, mixed, unclear | captive-bred |
| Life_history_notes | Key traits - socio-ecology/ life history if tested/ presented in paper | short text (e.g. caching intensity, sociality, foraging) | highly social, moderate caching |

**C. Sample & developmental staging**

| **Field** | **What it captures** | **Coding / format** | **Example** |
| --- | --- | --- | --- |
| N_total | total individuals tested | integer | 18 |
| N_by_age_class | breakdown per stage tested | structured text | Round 1: juvenile=8; sub-adult=6; adult=4; Round 2…; Observation: X age to X age |
| Age_reporting_type | how age is defined - based on analyses (continuous/ categorical) | calendar age, developmental stage, mixed, unclear | developmental stage |
| Age_min_max | numerical ages if given for testing | e.g. days, months, years | testing: 3–6 month; Observation: |
| Age_classes_used | age category tracking | nestling, fledgling, juvenile, sub-adult, adult, mixed ages | fledging then juvenile |
| Sex_distribution | sex info | e.g. M=9; F=7; unknown=2 | F=10; M=8 |
| Longitudinal_vs_cross_sectional | design | longitudinal (same individuals across life stages if specified), cross-sectional, repeated-measures, unclear, mixed | longitudinal, repeated measures |

**G. Tinbergen questions**

| **Field** | **What it captures** | **Coding / format** | **Example** |
| --- | --- | --- | --- |
| Developmental focus | does the study address development (age, experience, learning, life stage)? | yes, no, suggested, unclear | yes |
| Neuro/hormones/physiology | underlying mechanisms (physiology, neuro, hormones)? Is this specifically tested? | yes, no, suggested, unclear | yes |
| Fitness influence | adaptive value / fitness, survival, fitness-related proxy like body condition, body weight etc. - is this specifically tested? | yes, no, suggested, unclear | yes |
| Multi-species testing | multi-species testing in this study | yes, no, suggested, unclear | yes |

**D. Context & experience**

| **Field** | **What it captures** | **Coding / format** | **Example** |
| --- | --- | --- | --- |
| Housing_type | social housing outside of testing context | pair, family group, flock, single, mixed/varies | family group |
| Prior_experimental_experience | previous related testing | naïve, experienced, mixed, not reported | experienced |
| Relevant_ecological_experience | task-relevant reported history | free text | free-flying birds with natural caching opportunities |

**E. Cognitive domain, ability & task**

| **Field** | **What it captures** | **Coding / format** | **Example** |
| --- | --- | --- | --- |
| Cognitive_domain | broad domain | core/ foundational, social cognition, physical cognition, other | core/foundational, social |
| Specific_ability | finer-grained ability | e.g. inhibition, working memory, cognitive flexibility, causal reasoning, tool use, social learning, gaze following, object permanence - **use the terminology used in the paper** | inhibition |
| Task_paradigm_name | common label (use author named paradigm where given) | e.g. detour, go/no-go, A-not-B, reverse contingency, exchange task, change detection, match-to-sample, gaze-following - **use the terminology used in the paper** | detour |
| Task_brief_description | 1–2 sentence summary | free text | bird must walk around a transparent barrier to reach a visible reward |
| Task_domain_tag | is task tested while alone or in a social setting (conspecifics) | social, non-social, mixed | non-social |
| **Outcome_measures** | main dependent variables *(be specific -* ie. which behaviours measures; frequency/ latency/ duration/ proportion) | latency, % correct, trials_to_criterion, error_type, learning_rate, persistence, etc. | % correct; trials to criterion |
| Task_ecological_relevance | match to natural behaviour | high, moderate, low, unclear + comment | moderate; detours around obstacles are common |

**F. Developmental pattern & mechanisms**

| **Field** | **What it captures** | **Coding / format** | **Example** |
| --- | --- | --- | --- |
| Developmental_trajectory_type | overall pattern | improves/ increases with age, declines/ decrease with age, U-shaped, stable, no pattern, not analysed | improves with age |
| Individual_repeatability | any repeatability (statistically tested) | yes, no, not assessed, suggested (e.g. consistency), unclear | yes |
| Mechanistic_factors_tested | proximate influences tested | none, brain/neurology, hormones, nutrition, stress, social factors, training, other (multiple allowed) | social factors; training |
| Social_modulation_of_permformance | is social context experimentally manipulated as part of design? What effects of conspecific presence are there? | yes, no, unclear | yes |
| Environmental_modulation | ecological/ experience manipulation (not social context/ social environment) | enrichment, deprivation, wild vs captive, urban vs rural, none, etc. | wild vs captive |

**H. Quality & relevance indicators**

| **Field** | **What it captures** | **Coding / format** | **Example** |
| --- | --- | --- | --- |
| Sample_size_risk | rough power indicator - reported sample size for testing | very small (<10), small (10–19), moderate (20–39), large (≥40) | small |
| Age_definition_clarity | how clearly age/stage is described | clear, partial, poor | partial |
| Task_validity_comment | are tasks appropriate and validated? | free text + optional code: high, medium, low | Medium; performance confounded by neophobia |
| Overall_developmental_relevance | how useful for your review aims | high, moderate, low | high |
| Conservation_welfare_relevance | does this explicitly test the impact of cognition or behaviour on a conservation or welfare relevant outcome? E.g. fitness/ fitness-related proxies (wild and captive), enrichment | yes, no, implicit | yes |
| Main findings | overview of key findings from study | free text | offspring anti-predator behaviour increases with age |
| Comments | other text | free text | other notes, e.g. sex of subjects not reported, any areas where unsure |

## **References**

Antinucci, F., Spinozzi, G., Visalberghi, E., & Volterra, V. (1982). Cognitive development in a Japanese macaque (*Macaca fuscata*). *Annali Dell Istituto Superiore Di Sanità, 18*(2), 177–183. URL: https://www.researchgate.net/publication/16013358_Cognitive_development_in_a_Japanese_macaque_Macaca_fuscata

Auersperg, A. M., van Horik, J. O., Bugnyar, T., Kacelnik, A., Emery, N. J., & von Bayern, A. M. (2015). Combinatory actions during object play in psittaciformes (*Diopsittaca nobilis, Pionites melanocephala, Cacatua goffini*) and corvids (*Corvus corax, C. monedula, C. moneduloides*). *Journal of Comparative Psychology, 129*(1), 62.<https://doi.org/10.1037/a0038314>

Bebus, S. E., Small, T. W., Jones, B. C., Elderbrock, E. K., & Schoech, S. J. (2016). Associative learning is inversely related to reversal learning and varies with nestling corticosterone exposure. *Animal Behaviour, 111*, 251–260.<https://doi.org/10.1016/j.anbehav.2015.10.027>

Beck, K. B., Loretto, M.-C., & Bugnyar, T. (2020). Effects of site fidelity, group size and age on food-caching behaviour of common ravens, *Corvus corax*. *Animal Behaviour, 164*, 51–64. <https://doi.org/10.1016/j.anbehav.2020.03.015>

Boucherie, P. H., Blum, C., & Bugnyar, T. (2020). Effect of rearing style on the development of social behaviour in young ravens (*Corvus corax*). *Ethology, 126*(6), 595-609. <https://doi.org/10.1111/eth.13010>

Bräuer, J., Call, J., & Tomasello, M. (2005). All Great Ape Species Follow Gaze to Distant Locations and Around Barriers. *Journal of Comparative Psychology, 119*(2), 145–154. https://doi.org/10.1037/0735-7036.119.2.145

Braun, A., Walsdorff, T., Fraser, O. N., & Bugnyar, T. (2012). Socialized sub-groups in a temporary stable raven flock? *Journal of Ornithology, 153*(Suppl 1), 97-104. https://doi.org/10.1007/s10336-011-0810-2

Bugnyar, T., Stöwe, M., & Heinrich, B. (2007). The ontogeny of caching in ravens, *Corvus corax*. *Animal Behaviour, 74*(4), 757–767.<https://doi.org/10.1016/j.anbehav.2006.08.019>

Byrne, R., Mendl, M., Devereux, C., & Held, S. (2001). Behaviour of domestic pigs in a visual perspective taking task. *Behaviour, 138*(11-12), 1337–1354.<https://doi.org/10.1163/156853901317367627>

Call, J. (2001). Object permanence in orangutans (*Pongo pygmaeus*), chimpanzees (*Pan troglodytes*), and children (*Homo sapiens*). *Journal of Comparative Psychology, 115*(2), 159–171. https://doi.org/10.1037/0735-7036.115.2.159

Clary, D., Cheys, A., & Kelly, D. M. (2014). Pattern of visuospatial lateralization in two corvid species, black-billed magpies and Clark’s nutcrackers. *Behavioural Processes, 107*, 94–98. https://doi.org/10.1016/j.beproc.2014.07.020

Cornero, F. M., & Clayton, N. S. (2025). Object permanence in rooks (*Corvus frugilegus*): Individual differences and behavioral considerations. *Learning & Behavior, 53*(1), 93-113. https://doi.org/10.3758/s13420-024-00637-0

Damini, S., Blum, C. R., Sumasgutner, P., & Bugnyar, T. (2025). When to mob? plasticity of antipredator behavior in common ravens’ families (*Corvus corax*) across offspring development. *Animal Cognition, 28*(1), 55–55. https://doi.org/10.1007/s10071-025-01976-9

Davidson, G., Miller, R., Loissel, E., Cheke, L. G., & Clayton, N. S. (2017). The development of support intuitions and object causality in juvenile Eurasian jays (*Garrulus glandarius*). *Scientific Reports, 7*(1). https://doi.org/10.1038/srep40062

Dumas, C., & Doré, F. Y. (1989). Cognitive development in kittens (*Felis catus*): A cross-sectional study of object permanence. *Journal of Comparative Psychology, 103*(2), 191–200.<https://doi.org/10.1037/0735-7036.103.2.191>

Falótico, T., Bueno, C. Q., & Ottoni, E. B. (2021). Ontogeny and sex differences in object manipulation and probe tool use by wild tufted capuchin monkeys (*Sapajus libidinosus*). *American Journal of Primatology, 83*(5). https://doi.org/10.1002/ajp.23251

Ferrari, P., Kohler, E., Fogassi, L., & Gallese, V. (2000). The ability to follow eye gaze and its emergence during development in macaque monkeys. *Proceedings of the National Academy of Sciences of the United States of America, 97*(25), 13997–14002. https://doi.org/10.1073/pnas.250241197

Freeman, N. E., Norris, D. R., Sutton, A. O., Strickland, D., Kyser, T. K., & Newman, A. E. (2021). Early-life corticosterone body condition influence social status and survival in a food-caching passerine. *Integrative and Comparative Biology, 61*(1), 9-19.<https://doi.org/10.1093/icb/icab038>

Fuirst, M., Greer, J., & Bowman, R. (2020). Ontogeny of food‐caching site preferences in young Florida Scrub‐Jays: evidence of learning or changing social status? *Journal of Field Ornithology, 91*(3), 241–253.<https://doi.org/10.1111/jofo.12338>

Funk, M. S. (1996). Development of object permanence in the New Zealand parakeet (*Cyanoramphus auriceps*). *Animal Learning & Behavior, 24*(4), 375–383. https://doi.org/10.3758/bf03199009

Gagnon, S., & Doré, F. Y. (1994). Cross-sectional study of object permanence in domestic puppies (*Canis familiaris*). *Journal of Comparative Psychology, 108*(3), 220–232. https://doi.org/10.1037/0735-7036.108.3.220

Gallego-Abenza, M., Boucherie, P. H., & Bugnyar, T. (2022). Early social environment affects attention to social cues in juvenile common ravens, *Corvus corax*. *Royal Society Open Science, 9*(6). https://doi.org/10.1098/rsos.220132

Gallego‐Abenza, M., Loretto, M. C., & Bugnyar, T. (2020). Decision time modulates social foraging success in wild common ravens, *Corvus corax*. *Ethology, 126*(4), 413-422. https://doi.org/10.1111/eth.12986

Gallup, A. C., Schild, A. B., Ühlein, M. A., Bugnyar, T., & Massen, J. J. (2022). No evidence for contagious yawning in juvenile ravens (*Corvus corax*): an observational study. *Animals, 12*(11), 1357.<https://doi.org/10.3390/ani12111357>

Giret, N., Miklósi, Á., Kreutzer, M., & Bovet, D. (2008). Use of experimenter-given cues by African gray parrots (*Psittacus erithacus*). *Animal Cognition, 12*(1), 1–10.<https://doi.org/10.1007/s10071-008-0163-2>

Gómez, J. (2005). Species comparative studies and cognitive development. *Trends in Cognitive Sciences, 9*(3), 118–125. https://doi.org/10.1016/j.tics.2005.01.004

Greggor, A. L., Masuda, B., Flanagan, A. M., & Swaisgood, R. R. (2020). Age-related patterns of neophobia in an endangered island crow: implications for conservation and natural history. *Animal Behaviour, 160*, 61–68. https://doi.org/10.1016/j.anbehav.2019.12.002

Hare, B., Brown, M., Williamson, C., & Tomasello, M. (2002). The Domestication of Social Cognition in Dogs. *Science, 298*(5598), 1634-1636.<https://doi.org/10.1126/science.1072702>

Hare, B., Plyusnina, I., Ignacio, N., Schepina, O., Stepika, A., Wrangham, R., & Trut, L. (2005). Social Cognitive Evolution in Captive Foxes Is a Correlated By-Product of Experimental

Domestication. *Current Biology, 15*(3), 226–230.<https://doi.org/10.1016/j.cub.2005.01.040>

Hauser, M., Pearson, H., & Seelig, D. (2002). Ontogeny of tool use in cottontop tamarins, *Saguinus oedipus*: innate recognition of functionally relevant features. *Animal Behaviour, 64*(2), 299–311. https://doi.org/10.1006/anbe.2002.3068

Higuchi, H. (2008). Bait-fishing by the Green-backed Heron *Ardeola striata* in Japan. *IBIS, 128*(2), 285–290. https://doi.org/10.1111/j.1474-919x.1986.tb02677.x

Hoffmann, A., Rüttler, V., & Nieder, A. (2011). Ontogeny of object permanence and object tracking in the carrion crow, *Corvus corone*. *Animal Behaviour, 82*(2), 359–367. <https://doi.org/10.1016/j.anbehav.2011.05.012>

Holzhaider, J. C., Hunt, G. R., & Gray, R. D. (2010a). Social learning in New Caledonian crows. *Learning & Behavior, 38*(3), 206-219. https://doi.org/10.3758/LB.38.3.206

Holzhaider, J. C., Hunt, G. R., & Gray, R. D. (2010b). The development of pandanus tool manufacture in wild New Caledonian crows. *Behaviour*, *147*(5-6), 553-586.<https://doi.org/10.1163/000579510X12629536366284>

Holzhaider, J. C., Sibley, M. D., Taylor, A. H., Singh, P. J., Gray, R. D., & Hunt, G. R. (2011). The social structure of New Caledonian crows. *Animal Behaviour, 81*(1), 83-92. https://doi.org/10.1016/j.anbehav.2010.09.015

Hunt, G. R., Lambert, C., & Gray, R. D. (2007). Cognitive requirements for tool use by New Caledonian crows (*Corvus moneduloides*). *New Zealand Journal of Zoology, 34*(1), 1-7. <https://doi.org/10.1080/03014220709510058>

Jaakkola, K. (2014). Do animals understand invisible displacement? A critical review. *Journal of Comparative Psychology, 128*(3), 225. <https://doi.org/10.1037/a0035675>

Jacobs, I., Kabadayi, C., & Osvath, M. (2019). The Development of Sensorimotor Cognition in Common Ravens (*Corvus corax*) and its Comparative Evolution. *Animal Behavior and Cognition, 6*(3), 194–212. https://doi.org/10.26451/abc.06.03.04.2019

Jaime, M., Lopez, J. P., & Lickliter, R. (2009). Bobwhite quail (*Colinus virginianus*) hatchlings track the direction of human gaze. *Animal Cognition, 12*(4), 559–565. https://doi.org/10.1007/s10071-009-0214-3

Kaminski, J., Riedel, J., Call, J., & Tomasello, M. (2005). Domestic goats, *Capra hircus*, follow gaze direction and use social cues in an object choice task. *Animal Behaviour, 69*(1), 11–18.<https://doi.org/10.1016/j.anbehav.2004.05.008>

Kehmeier, S., Schloegl, C., Isabella, & Weiß, B. M. (2011). Early development of gaze following into distant space in juvenile Greylag geese (*Anser anser*). *Animal Cognition, 14*(4), 477–485. https://doi.org/10.1007/s10071-011-0381-x

Kenward, B., Rutz, C., Weir, A. A. S., & Kacelnik, A. (2006). Development of tool use in New Caledonian crows: inherited action patterns and social influences. *Animal Behaviour, 72*(6), 1329–1343.<https://doi.org/10.1016/j.anbehav.2006.04.007>

Kenward, B., Schloegl, C., Rutz, C., Weir, A. A., Bugnyar, T., & Kacelnik, A. (2011). On the evolutionary and ontogenetic origins of tool-oriented behaviour in New Caledonian crows (*Corvus moneduloides*). *Biological Journal of the Linnean Society, 102*(4), 870-877. https://doi.org/10.1111/j.1095-8312.2011.01613.x

Kenward, B., Weir, A. A., Rutz, C., & Kacelnik, A. (2005). Tool manufacture by naive juvenile crows. *Nature, 433*(7022), 121-121. https://doi.org/10.1038/433121a

Loretto, M.-C., Schuster, R., Federspiel, I. G., Heinrich, B., & Bugnyar, T. (2020). Contextual imitation in juvenile common ravens, *Corvus corax*. *Animal Behaviour, 163*, 127–134.<https://doi.org/10.1016/j.anbehav.2020.03.007>

Mann, J., Sargeant, B. L., Watson-Capps, J. J., Gibson, Q. A., Heithaus, M. R., Connor, R. C., & Patterson, E. (2008). Why Do Dolphins Carry Sponges? *PLOS ONE, 3*(12), e3868. https://doi.org/10.1371/journal.pone.0003868

Mannu, M., & Ottoni, E. B. (2009). The enhanced tool-kit of two groups of wild bearded capuchin monkeys in the Caatinga: tool making, associative use, and secondary tools. *American Journal of Primatology*, 71(3), 242–251. https://doi.org/10.1002/ajp.20642

Mates, E. A., Tarter, R. R., Ha, J. C., Clark, A. B., & McGowan, K. J. (2014). Acoustic profiling in a complexly social species, the American crow: caws encode information on caller sex, identity and behavioural context. *Bioacoustics, 24*(1), 63–80.<https://doi.org/10.1080/09524622.2014.933446>

Meulman, E. J., Seed, A. M., & Mann, J. (2013). If at first you don't succeed… Studies of ontogeny shed light on the cognitive demands of habitual tool use. *Philosophical Transactions of the Royal Society B: Biological Sciences, 368*(1630), 20130050. https://doi.org/10.1098/rstb.2013.0050

Miller, R., Boeckle, M., Ridgway, S., Richardson, J., Uhl, F., Bugnyar, T., & Schwab, C. (2025). Social attention across development in common ravens and carrion/hooded crows. *Animal Behaviour, 220*, 123038.<https://doi.org/10.1016/j.anbehav.2024.11.016>

Miller, R., Bugnyar, T., Pölzl, K., & Schwab, C. (2015). Differences in exploration behaviour in common ravens and carrion crows during development and across social context. *Behavioral Ecology and Sociobiology, 69*(7), 1209-1220. https://doi.org/10.1007/s00265-015-1935-8

Miller, R., Laskowski, K. L., Schiestl, M., Bugnyar, T., & Schwab, C. (2016). Socially driven consistent behavioural differences during development in common ravens and carrion crows. *PLOS ONE, 11*(2), e0148822.<https://doi.org/10.1371/journal.pone.0148822>

Musgrave, S., Lonsdorf, E., Morgan, D., & Sanz, C. (2020). The ontogeny of termite gathering among chimpanzees in the Goualougo Triangle, Republic of Congo. *American Journal of Physical Anthropology, 174*(2).<https://doi.org/10.1002/ajpa.24125>

Neiworth, J. J., Burman, M. A., Basile, B. M., & Lickteig, M. T. (2002). Use of experimenter-given cues in visual co-orienting and in an object-choice task by a New World monkey species, Cotton Top Tamarins (*Saguinus oedipus*). *Journal of Comparative Psychology, 11*6(1), 3–11. https://doi.org/10.1037/0735-7036.116.1.3

Obozova, T. A., Smirnova, A. A., & Zorina, Z. A. (2018). Early Ontogeny of the Behavior of Young in Large-Billed Crows (*Corvus macrorhynchos*) in Their Natural Habitat. *Biology Bulletin, 45*(8), 794-802. https://doi.org/10.1134/S1062359018080125

Parron, C., & Meguerditchian, A. (2016). Gaze following in baboons (*Papio anubis*): juveniles adjust their gaze and body position to human’s head redirections. *American Journal of Primatology, 78*(12), 1265–1271. https://doi.org/10.1002/ajp.22580

Payne, S. F., & Jameson, R. J. (1984). Early Behavioral Development of the Sea Otter, *Enhydra lutris*. *Journal of Mammalogy, 65*(3), 527–531. https://doi.org/10.2307/1381114

Pepperberg, I. M., Willner, M. R., & Gravitz, L. B. (1997). Development of Piagetian object permanence in grey parrot (*Psittacus erithacus*). *Journal of Comparative Psychology, 111*(1), 63–75.<https://doi.org/10.1037/0735-7036.111.1.63>

Pollok, B., Prior, H., & Güntürkün, O. (2000). Development of object permanence in food-storing magpies (*Pica pica*). *Journal of Comparative Psychology, 114*(2), 148–157. https://doi.org/10.1037/0735-7036.114.2.148

Range, F., & Virányi, Z. (2011). Development of gaze following abilities in wolves (*Canis lupus*). *PLOS ONE, 6*(2), e16888.<https://doi.org/10.1371/journal.pone.0016888>

Rosa Salva, O., Regolin, L., & Vallortigara, G. (2007). Chicks discriminate human gaze with their right hemisphere. *Behavioural Brain Research, 177*(1), 15–21.<https://doi.org/10.1016/j.bbr.2006.11.020>

Salwiczek, L. H., Emery, N. J., Schlinger, B., & Clayton, N. S. (2009). The development of caching and object permanence in western scrub-jays (*Aphelocoma californica*): Which emerges first? *Journal of Comparative Psychology, 123*(3), 295–303.<https://doi.org/10.1037/a0016303>

Scerif, G., Gomez, J.-C., & Byrne, R. W. (2004). What do Diana monkeys know about the focus of attention of a conspecific? *Animal Behaviour, 68*(6), 1239–1247. https://doi.org/10.1016/j.anbehav.2004.01.011

Scheid, C., & Bugnyar, T. (2008). Short-term observational spatial memory in Jackdaws (*Corvus monedula*) and Ravens (*Corvus corax*). *Animal Cognition, 11*(4), 691-698.<https://doi.org/10.1007/s10071-008-0160-5>

Schino, G., Spinozzi, G., & Berlinguer, L. (1990). Object concept and mental representation in *Cebus apella* and *Macaca fascicularis*. *Primates,* 31(4), 537–544. https://doi.org/10.1007/bf02382536

Schloegl, C., Kotrschal, K., & Bugnyar, T. (2007). Gaze following in common ravens, *Corvus corax*: ontogeny and habituation. *Animal Behaviour, 74*(4), 769–778.<https://doi.org/10.1016/j.anbehav.2006.08.017>

Schuppli, C., Van Cauwenberghe, A., Mirta Setia, T., & Haun, D. (2021). The ontogeny of exploratory object manipulation behaviour in wild orangutans. *Evolutionary Human Sciences, 3*, 1–32. https://doi.org/10.1017/ehs.2021.34

Schwab, C., Bugnyar, T., & Kotrschal, K. (2008). Preferential learning from non-affiliated individuals in jackdaws (*Corvus monedula*). *Behavioural Processes, 79*(3), 148-155. https://doi.org/10.1016/j.beproc.2008.07.002

Simpson, J., & O’Hara, S. J. (2018). Gaze following in an asocial reptile (*Eublepharis macularius*). *Animal Cognition, 22*(2), 145–152.<https://doi.org/10.1007/s10071-018-1230-y>

Smith, B. P., & Litchfield, C. A. (2009). Dingoes (*Canis dingo*) can use human social cues to locate hidden food. *Animal Cognition, 13*(2), 367–376. https://doi.org/10.1007/s10071-009-0287-z

Spinozzi, G., & Natale, F. (2019). Early Sensorimotor Development in Gorilla. *In Cognitive Structure and Development in Nonhuman Primates* (pp. 21–38). Psychology Press.<https://doi.org/10.4324/9781315808055-5>

Stafford, B. L., Balda, R. P., & Kamil, A. C. (2006). Does seed‐caching experience affect spatial memory performance by pinyon jays? *Ethology, 112*(12), 1202-1208.<https://doi.org/10.1111/j.1439-0310.2006.01279.x>

Stöwe, M., Bugnyar, T., Loretto, M. C., Schloegl, C., Range, F., & Kotrschal, K. (2006). Novel object exploration in ravens (*Corvus corax*): effects of social relationships. *Behavioural Processes, 73*(1), 68-75.<https://doi.org/10.1016/j.beproc.2006.03.015>

Szabó, E., Chiandetti, C., Téglás, E., Versace, E., Csibra, G., Kovács, Á. M., & Vallortigara, G. (2022). Young domestic chicks spontaneously represent the absence of objects. *ELife, 11*. https://doi.org/10.7554/elife.67208

Tan, A. W. (2017). From play to proficiency: The ontogeny of stone-tool use in coastal-foraging long-tailed macaques (*Macaca fascicularis*) from a comparative perception-action perspective. *Journal of Comparative Psychology, 131*(2), 89. https://doi.org/10.1037/com0000068

Tebbich, S., Seed, A. M., Emery, N. J., & Clayton, N. S. (2007). Non-tool-using rooks, *Corvus frugilegus*, solve the trap-tube problem. *Animal Cognition, 10*(2), 225-231.<https://doi.org/10.1007/s10071-006-0061-4>

Tebbich, S., Taborsky, M., Fessl, B., & Blomqvist, D. (2001). Do woodpecker finches acquire tool-use by social learning? Proceedings of the Royal Society of London. *Series B: Biological Sciences, 268*(1482), 2189–2193. https://doi.org/10.1098/rspb.2001.1738

Teufel, C., Gutmann, A., Pirow, R., & Fischer, J. (2010). Facial expressions modulate the ontogenetic trajectory of gaze-following among monkeys. *Developmental Science, 13*(6), 913–922. https://doi.org/10.1111/j.1467-7687.2010.00956.x

Tomasello, M., Hare, B., & Fogleman, T. (2001). The ontogeny of gaze following in chimpanzees, *Pan troglodytes*, and rhesus macaques, *Macaca mulatta*. *Animal Behaviour, 61*(2), 335-343.<https://doi.org/10.1006/anbe.2000.1598>

Ujfalussy, D. J., Miklósi, Á., & Bugnyar, T. (2012). Ontogeny of object permanence in a non-storing corvid species, the jackdaw (*Corvus monedula*). *Animal Cognition, 16*(3), 405–416.<https://doi.org/10.1007/s10071-012-0581-z>

Uzgiris, I. C., & Hunt, J. (1975). *Assessment in infancy: Ordinal scales of psychological development*. University of Illinois Press.

von Bayern, A., de Kort, S., Clayton, N., & Emery, N. (2007). The role of food-and object-sharing in the development of social bonds in juvenile jackdaws (*Corvus monedula*). *Behaviour, 144*(6), 711-733.<https://doi.org/10.1163/156853907781347826>

Wang, L., Luo, Y., Abudusaimaiti Maierdiyali, Chang, H., Ullah, S., & Li, Z. (2021). Azure-winged Magpies *Cyanopica cyanus* passed the tasks on the Uzgiris-Hunt scale of object permanence. *Journal Für Ornithologie, 162*(2), 605–613. https://doi.org/10.1007/s10336-021-01856-2

Wang, X., Yu, F., Si, H., & Li, Z. (2023). Vocal repertoire of Azure-winged Magpies (*Cyanopica cyanus*): A context-associated communication system. *Avian Research, 14*, 100089.<https://doi.org/10.1016/j.avrs.2023.100089>

Wascher, C. A., & Youngblood, M. (2025). Vocal efficiency in crows. *Animal Cognition, 28*(1), 75. https://doi.org/10.1007/s10071-025-01985-8

Wenig, K., Boucherie, P. H., & Bugnyar, T. (2021). Early evidence for emotional play contagion in juvenile ravens. *Animal Cognition, 24*(4), 717-729.<https://doi.org/10.1007/s10071-020-01466-0>

Wise, K. L., Wise, L. A., & Zimmermann, R. R. (1974). Piagetian object permanence in the infant rhesus monkey. *Developmental Psychology, 10*(3), 429–437. <https://doi.org/10.1037/h0036405>

Wood, S., Moriarty, K. M., Gardner, B. T., & Gardner, R. A. (1980). Object permanence in child and chimpanzee. *Animal Learning & Behavior, 8*(1), 3–9. https://doi.org/10.3758/bf03209723

Zandberg, L., Jolles, J. W., Boogert, N. J., & Thornton, A. (2014). Jackdaw nestlings can discriminate between conspecific calls but do not beg specifically to their parents. *Behavioral Ecology, 25*(3), 565–573. https://doi.org/10.1093/beheco/aru026

Zeiträg, C., Jensen, T. R., & Osvath, M. (2022). Gaze following: A socio-cognitive skill rooted in deep time. *Frontiers in Psychology*, *13*, 950935.<https://doi.org/10.3389/fpsyg.2022.950935>

Zeiträg, C., & Osvath, M. (2023). Differential responses to con- and allospecific visual cues in juvenile ravens (*Corvus corax*): the ontogeny of gaze following and social predictions. *Animal Cognition, 26*.<https://doi.org/10.1007/s10071-023-01772-3>

Zucca, P., Milos, N., & Vallortigara, G. (2007). Piagetian object permanence and its development in Eurasian jays (*Garrulus glandarius*). *Animal Cognition*, *10*(2), 243-258.<https://doi.org/10.1007/s10071-006-0063-2>
